# Multisystem mitochondrial disease caused by a rare m.10038G>A mitochondrial tRNA^Gly^ (*MT-TG*) variant

**DOI:** 10.1212/NXG.0000000000000413

**Published:** 2020-03-18

**Authors:** Olivia V. Poole, Alejandro Horga, Steven A. Hardy, Enrico Bugiardini, Cathy E. Woodward, Iain P. Hargreaves, Ashirwad Merve, Rosaline Quinlivan, Robert W. Taylor, Michael G. Hanna, Robert D.S. Pitceathly

**Affiliations:** From the Department of Neuromuscular Diseases (O.V.P., A.H., E.B., R.Q., M.G.H., R.D.S.P.), UCL Queen Square Institute of Neurology and The National Hospital for Neurology and Neurosurgery, London, United Kingdom; Wellcome Centre for Mitochondrial Research (S.A.H., R.W.T.), Translational and Clinical Research Institute, Newcastle University, Newcastle Upon Tyne; Neurogenetics Unit (C.E.W.), and Neurometabolic Unit (I.P.H.), The National Hospital for Neurology and Neurosurgery; Division of Neuropathology (A.M.), UCL Queen Square Institute of Neurology; Department of Histopathology (A.M.), Camelia Botnar Laboratory, Great Ormond Street Hospital; and Dubowitz Neuromuscular Centre (R.Q.), Great Ormond Street Hospital, London, United Kingdom.

Most pathogenic mitochondrial DNA (mtDNA) variants occur in the 22 mtDNA-encoded tRNA (mt-tRNA) genes. However, despite more than 270 reported mt-tRNA gene mutations, only 5 reside within mt-tRNA^Gly^ (*MT-TG*).^1^ We report a rare *MT-TG* variant and evaluate this, in addition to all previously reported *MT-TG* variants, against the published criteria used to help determine the pathogenicity of the mt-tRNA variants.^2^

## Case report

A 39 year old woman, born to nonconsanguineous parents, was reviewed in a specialist mitochondrial disorders clinic. She presented with hearing loss in her late teens followed by visual impairment, with bilateral cataracts, retinal dystrophy, and subsequent bilateral retinal detachments in her twenties; hypothyroidism in her thirties; and secondary amenorrhea. Clinical examination was otherwise normal, apart from short stature. There was no family history of neuromuscular or neurologic disease. Blood tests, including creatine kinase, plasma amino acids, acylcarnitine profile, very long chain fatty acids, and white cell enzymes, were normal. Plasma lactate was elevated (3.70 mmol/L, reference range 0.5–2.2 mmol/L). Nerve conduction studies and EMG showed no evidence of generalized myopathy or large fiber neuropathy. Histochemical analyses of muscle tissue revealed ragged-red and cytochrome *c* oxidase (COX) deficient fibers (figure A). Spectrophotometric determination of mitochondrial respiratory chain enzyme activities as a ratio to citrate synthase activity^3^ confirmed decreased activities of complexes I (0.076, reference range 0.104–0.268) and IV (0.006, reference range 0.014–0.034). Analysis of the next generation sequencing data (Ilumina MiSeq) of the entire mitochondrial genome extracted from the muscle^3^ revealed a rare m.10038G>A variant (GenBank reference accession number: NC_012920.1) in *MT-TG* (figure B) that was present at variable heteroplasmy levels across tissue types: 15% blood, 40% urinary epithelial cells, and 92% skeletal muscle. Maternal transmission was confirmed: 3% mutant load was present in the mother's urinary epithelial cells (methodology detects heteroplasmy levels ≥1%). Heteroplasmy levels within individual laser-captured COX-positive and COX-deficient muscle fibers were quantified by pyrosequencing.^4^ Single fiber segregation studies confirmed a higher mutation load in COX-deficient (mean 95.30% ± 0.50%, SD, n = 27) compared with COX-positive fibers (mean 78.92% ± 4.43%, SD, n = 26; *p* = 0.0005, figure C).

**Figure:**
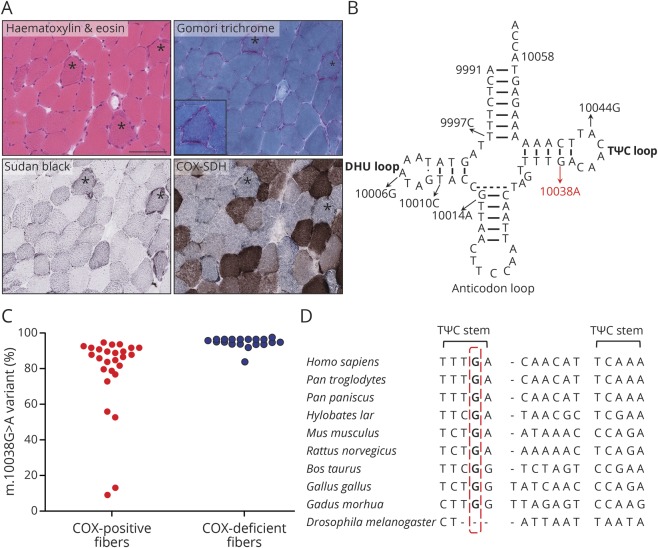
Characterization of a rare pathogenic *MT-TG* variant (A) Staining of the muscle sections with haematoxylin and eosin demonstrated ragged-red fibers (*) which were confirmed in the Gomori trichrome preparation (*) with the inset showing high magnification of left top fiber. A number of fibers, often with ragged-red morphology, showed a mild increase in the number of lipid droplets with Sudan black (*). Sequential COX and SDH histochemistry demonstrated frequent COX-deficient fibers, some of which had a ragged red–like appearance (*). The bar represents 100 µm for all stains with the inset 50 μm. (B) Two-dimensional cloverleaf structure of mitochondrial DNA-encoded tRNA^Gly^. Black arrows indicate previously reported pathogenic variants. Red arrow indicates the rare variant identified in our patient. (C) Single muscle fiber segregation studies. COX-deficient fibers, (blue circles) harbor a higher mutational load compared with COX-positive fibers (red circles). (D) Evolutionary conservation of the m.10038G residue across species. COX = cytochrome *c* oxidase; SDH = succinate dehydrogenase.

## Discussion

The m.10038G nucleotide is highly conserved across species (figure D, figure e-1, links.lww.com/NXG/A248) and the G>A transition at this location disrupts a C-G Watson-Crick base pair in the TΨC stem of the tRNA molecule. The m.10038G>A variant is rare (absent from 3,450 in-house and 48,882 GenBank^1^ sequences) and detectable at variable heteroplasmy levels across different tissues, with the highest levels in the postmitotic muscle. Histochemical and biochemical evidence of reduced complex I and IV activities in the muscle is supportive of impaired mitochondrial protein synthesis, whereas single fiber studies confirmed segregation of the COX defect with higher mutant levels. Consequently, the mutation would be considered “definitely pathogenic” based on the accepted criteria for assigning pathogenicity to tRNA mutations (table e-1, links.lww.com/NXG/A249).^2^

Review of the 5 previously reported *MT-TG* variants according to these criteria (table e-1, links.lww.com/NXG/A249) reveals that only 2 variants; m.9997T>C and m.10010T>C, should be considered “definitely pathogenic.” Although the m.9997T>C variant has only been reported in one family, *trans*mitochondrial cybrid studies support its pathogenic effects. The m.10010T>C variant has been reported a number of times, and its pathogenicity has been confirmed using single fiber segregation studies. The m.10006A>G variant has only been reported in individuals harboring additional mtDNA variants, and thus may represent a benign polymorphism or be insufficient to cause disease in isolation. Current evidence suggests that the m.10014A>G variant is a benign polymorphism. Finally, although the scoring system indicates that the m.10044A>G variant is “possibly pathogenic,” it has been detected in healthy controls, and thus may represent a haplogroup specific polymorphism.^5^

It remains unclear why pathogenic variants occur more frequently in specific mt-tRNA genes. Possible explanations include intensive investigation of mt-tRNAs in which pathogenic variants have previously been identified, and survivor bias and the absence of maternal transmission of mutations in mt-tRNA genes linked with severe biochemical deficiencies, with isolated cases dissuading clinicians from further investigation of an underlying mitochondrial disorder.^6^ Variants in mt-tRNA^Gly^ affecting its canonical or noncanonical functions may also potentially be better tolerated than other mt-tRNAs or, conversely, be severely deleterious and embryonic lethal.

The detection of the rare mt-tRNA gene variants in suspected mitochondrial disease has become more commonplace, given the widespread availability of whole mtDNA high throughput sequencing. Furthermore, whole genome sequencing, which includes capture and deep sequencing of the mitochondrial genome, is identifying mtDNA variants in those in whom mitochondrial disease may not previously have been considered. However, despite advances in the DNA sequencing technology, the challenge of assigning pathogenicity to the rare mtDNA variants remains. It is therefore crucial that pathogenic variants are reported after confirmation using “gold-standard” techniques, for example, single fiber or *trans*mitochondrial cybrid studies, given the implications to genetic counseling and the available reproductive options for mtDNA mutations, including mitochondrial donation. Moreover, generating a comprehensive data set for “definitely pathogenic” mt-tRNA variants would potentially advance the understanding of the molecular mechanisms underpinning the susceptibility of individual genes to deleterious variants and facilitate the development of targeted therapies to treat this group of disorders.
